# First trimester maternal serum PAPP-A and free β-hCG levels and risk of SGA or LGA in women with and without GDM

**DOI:** 10.1186/s12884-024-06786-4

**Published:** 2024-09-06

**Authors:** Tiina Kantomaa, Marja Vääräsmäki, Mika Gissler, Markku Ryynänen, Jaana Nevalainen

**Affiliations:** 1 Department of Obstetrics and Gynecology, Oulu University Hospital, Oulu, Finland; 2 Research Unit of Clinical Medicine and Medical Research Center, University of Oulu, Oulu, Finland; 3 Information Department, THL Finnish Institute for Health and Welfare, Helsinki, Finland; 4 Department of Molecular Medicine and Surgery, Karolinska Institute, 171 76 Stockholm, Sweden

**Keywords:** First trimester screening, Free β-human chorionic gonadotropin (fβ-hCG), Gestational diabetes mellitus (GDM), Large for gestational age (LGA), Small for gestation age (SGA), Pregnancy associated plasma protein-A (PAPP-A)

## Abstract

**Background:**

Maternal gestational diabetes (GDM), small (SGA) and large (LGA) for gestational age neonates are associated with increased morbidity in both mother and child. We studied how different levels of first trimester pregnancy associated plasma protein-A (PAPP-A) and free beta human chorionic gonadotropin (fβ-hCG) were associated with SGA and LGA in GDM pregnancies and controls.

**Methods:**

Altogether 23 482 women with singleton pregnancies participated in first trimester combined screening and delivered between 2014 and 2018 in Northern Finland and were included in this retrospective case-control study. Women with GDM (*n* = 4697) and controls without GDM (*n* = 18 492) were divided into groups below 5th and 10th or above 90th and 95th percentile (pc) PAPP-A and fβ-hCG MoM levels. SGA was defined as a birthweight more than two standard deviations (SD) below and LGA more than two SDs above the sex-specific and gestational age-specific reference mean. Odds ratios were adjusted (aOR) for maternal age, BMI, ethnicity, IVF/ICSI, parity and smoking.

**Results:**

In pregnancies with GDM the proportion of SGA was 2.6% and LGA 4.5%, compared to 3.3% (*p* = 0.011) and 1.8% (*p* < 0.001) in the control group, respectively. In ≤ 5th and ≤ 10th pc PAPP-A groups, aORs for SGA were 2.7 (95% CI 1.5–4.7) and 2.2 (95% CI 1.4–3.5) in the GDM group and 3.8 (95% CI 3.0–4.9) and 2.8 (95% CI 2.3–3.5) in the reference group, respectively. When considering LGA, there was no difference in aORs in any high PAPP-A groups. In the low ≤ 5 percentile fβ-hCG MoM group, aORs for SGA was 2.3 (95% CI 1.8–3.1) in the control group. In fβ-hCG groups with GDM there was no association with SGA and the only significant difference was ≥ 90 percentile group, aOR 1.6 (95% CI 1.1–2.5) for LGA.

**Conclusion:**

Association with low PAPP-A and SGA seems to be present despite GDM status. High PAPP-A levels are not associated with increased LGA risk in women with or without GDM. Low fβ-hCG levels are associated with SGA only in non-GDM pregnancies.

**Supplementary Information:**

The online version contains supplementary material available at 10.1186/s12884-024-06786-4.

## Background

Gestational diabetes mellitus (GDM) is defined as the onset of glucose intolerance that is identified for the first-time during pregnancy [1]. GDM is associated with increased risks for the mother and the newborn, including pre-eclampsia, premature delivery, large for gestational age (LGA) birthweight, shoulder dystocia and birth trauma, increased risk for caesarean section and neonatal morbidity [2]. In the long term, the mother is at increased risk for recurrent GDM, subsequent type 2 diabetes, metabolic syndrome and cardiovascular diseases [3–5]. Antenatal predisposing to maternal GDM is associated with subsequent impaired glucose intolerance and obesity [3, 6].

Pregnancy associated plasma protein-A (PAPP-A) is an insulin-like growth factor binding protein protease (IGFBP) with an important role in fetal growth and development [7]. Low level of PAPP-A is an indicator of placental pathogenesis and has been associated with several pregnancy complications such as chromosomal abnormalities, preeclampsia, and small for gestational age (SGA) newborns [8]. The association of PAPP-A with metabolic diseases, such as GDM, has also been found; in some earlier studies, low PAPP-A values have been associated with GDM, [9–15] but not in all [16–19]. The role of PAPP-A in the prediction of GDM seems questionable [11, 12, 20, 21]. Low PAPP-A MoM is a risk factor for SGA, [8, 22, 23] whereas high PAPP-A MoM is a protective factor for SGA and was also associated with LGA in earlier studies [24–26]. High PAPP-A has been found to predict LGA [10]. The role of PAPP-A seems not to be restricted only to pregnancy as low first trimester PAPP-A levels have also been associated with the risk of development of metabolic and cardiovascular diseases such as diabetes later in life in mothers and short stature in offspring [27].

Free β-human chorionic gonadotropin (fβ-hCG) is produced by trophoblasts and is a pregnancy-specific hormone that regulates placental development, uterine and fetal growth [28]. Together with PAPP-A, fβ-hCG is measured from maternal serum in the first trimester as part of combined screening program for chromosomal abnormalities as fβ-hCG values are increased in trisomy 21 pregnancies and decreased in trisomy 18 and 13 pregnancies [29]. Low fβ-hCG concentration is associated with low birthweight, preterm birth and low Apgar scores [23, 30–32]. Previous studies have reported contradictory results on the association between high MoM levels of fβ-hCG and SGA. The high fβ-hCG MoM levels seemed to be a protective factor for GDM and preterm birth in previous studies [32–35]. 

First trimester maternal serum markers PAPP-A and fβ-hCG have been shown to be associated with several pregnancy complications that affect fetal weight. The purpose of this study was to investigate the role of abnormal maternal serum biochemistry in fetal growth in GDM and non-GDM pregnancies. This is interesting as low PAPP-A is associated with the risk of SGA newborns but also with the risk of GDM which on the contrary is associated with LGA newborns. Our hypothesis in this study was that gestational diabetes would affect fetal growth more than abnormal first trimester biochemistry. Our primary aim was to determine whether low PAPP-A MoM in women with GDM is associated with an increased risk of SGA or LGA. We also investigated whether high PAPP-A MoM levels as such are associated with LGA. Furthermore, we examined the association between fβ-hCG MoM levels and the risk of SGA and LGA in pregnancies with or without GDM.

## Materials and methods

This was a retrospective case-control study that comprised 23 482 women with singleton pregnancies. Women voluntarily attended the first trimester combined screening in the Oulu University Hospital region and delivered between January 1st, 2014, and December 31st, 2018, in delivery units in Finland. The national screening program was performed according to the guidelines of the Finnish Ministry of Social Affairs and Health. The Finnish Medical Birth Register (MBR), which includes the data on all births of liveborn and stillborn infants ≥ 22 + 0 gestational weeks (gw) or ≥ 500 g in Finland, was utilized for data on all pregnancy outcomes. Pregnancies with results of termination or miscarriage were not included. Also, pregnancies with trisomy 21 (ICD-10 code Q90 *n* = 11), 18 and 13 (ICD-10 code Q91 *n* = 2) were excluded from the data.

The measurements of maternal serum PAPP-A and fβ-hCG levels were made between gw 9 + 0 and 13 + 6 and quantitated with time-resolved immunofluorometric assays on an automatic immunoanalyzer (AutoDELFIA^®^, PerkinElmer Wallac, Finland) at an accredited Nordlab laboratory (Oulu, Finland). The results were given as multiple of medians (MoM). [36] Pregnancy associated plasma protein-A MoM values were adjusted with gestational age, maternal age and weight, smoking status, ethnicity (Caucasian, Asian, Afro-Caribbean, Oriental and other) and diabetes. Nuchal translucency (NT) was measured by a trained sonographer or a specialist doctor of obstetrics and gynecology between gw 11 + 0 and 13 + 6. The Fetal Medicine Foundation protocol was used for NT measurements [37]. Gestational age was confirmed with ultrasound. It was corrected if fetal crown-rump length (CRL) differed from the assumed gestational age by five days or more.

Maternal age was defined as the mother’s age at the first antenatal visit. Maternal body mass index (BMI) was calculated as the mother’s pre-pregnancy weight divided by the square of her height (kg/m^2^). Any smoking occurring during the pregnancy was defined as smoking. Data were obtained from screening data and MBR.

The 75 g oral glucose tolerance test (OGTT) was routinely performed in all pregnancies between the 24th and 28th gw. However, exceptions were made for primiparas under 25 years of age with normal weight (BMI 18.5–24.9 kg/m^2^) and no family history of type 2 diabetes, as well as multiparas under 40 years of age with normal weight (BMI under 25 kg/m^2^) and no previous instances of GDM or LGA babies. In high-risk populations (BMI ≥ 35 kg/m^2^, prior GDM, family history of type 2 diabetes, polycystic ovary syndrome, glucosuria in early pregnancy, oral corticosteroid medication) OGTT was performed already between the 12th and 16th gw [38]. If the early OGTT was normal, it was re-tested between the 24th and 28th gw. The test was considered abnormal if glucose concentrations in OGTT were in venous plasma after a 12-hour overnight fast ≥ 5.3 mmol/l, at 1 h ≥ 10.0 mmol/l and 2 hours ≥ 8.6 mmol/l after the glucose load. The diagnosis of GDM was set after one or several abnormal glucose concentrations. In the present study the diagnosis of GDM was based on an abnormal OGTT result and/or ICD-10 codes O24.4 and/or O24.9 according to MBR. Every woman gets lifestyle counseling (weight gain, nutrition, physical exercise, and blood pressure recommendations) during pregnancy from the primary health care on their first antenatal visit. Weight gain recommendations are based on guidelines of Institute of Medicine [39]. If a woman is diagnosed GDM, she will have diabetic nutritional recommendations and counseling from primary health care according to national guidelines (Finnish Current Care Guidelines of Gestational Diabetes) [38]. These instructions follow the general nutrition recommendations during pregnancy and nutritional treatment recommendations for diabetes. After the GDM diagnosis and counseling, women began glucose self-monitoring. Medical treatment (insulin and/or metformin) was considered if plasma glucose concentrations, despite detailed dietary counseling, repeatedly exceeded the target levels (< 5.5 mmol/L fasting, < 7.8 mmol/L 1 h postprandial) [38, 40]. 

**Fig. 1:**
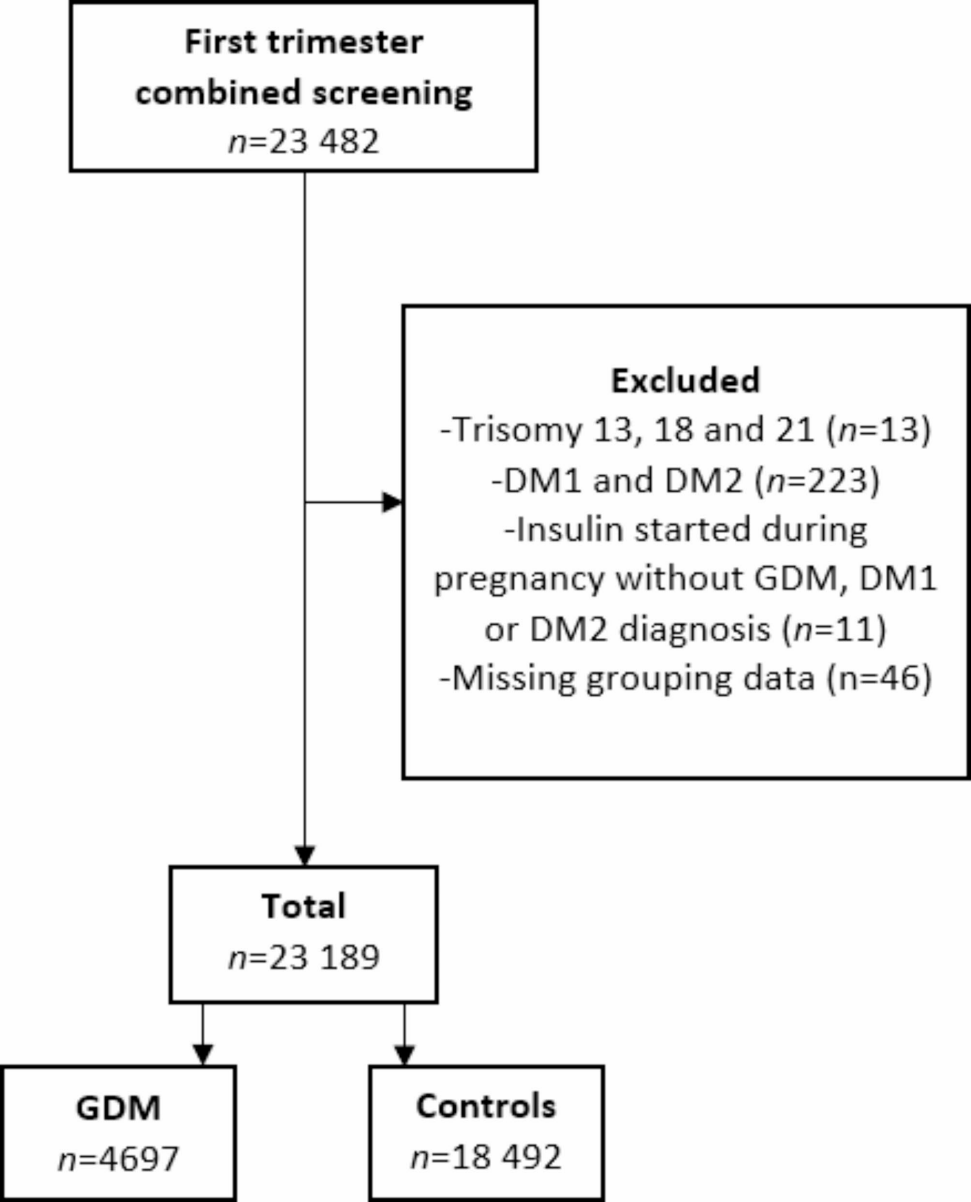
Study flow chart

The control population was defined as participants with no diagnosis of GDM. Women with pre-pregnancy diabetes (type 1 or type 2, ICD-10 codes E10, E11, O24.0, and/or O24.1) were excluded (*n* = 223). Four cases were excluded because of missing data and 11 because insulin was started during pregnancy without a diagnosis of GDM, diabetes type 1 or type 2 according to MBR. The flow chart describes the inclusion and exclusion of participants (Fig. 1).

According to the International Societies of Pediatric Endocrinology and the Growth Hormone Research Society small for gestational age (SGA) was defined as a birthweight more than two standard deviations (SDs) below and large for gestational age (LGA) more than two SDs above the sex-specific and gestational age-specific reference mean [41]. Appropriate for gestational age (AGA) was used as a reference value. Data to define SGA, AGA and LGA were missing in 42 pregnancies, and therefore those cases were excluded from the study.

Women with GDM and controls were divided into groups according to PAPP-A and fβ-hCG MoM values. Limits of ≤ 5th percentile (pc), ≤ 10th pc, ≥ 90th pc and ≥ 95th pc were used as grouping values.

### Statistical analysis

Statistical analysis was made with IBM SPSS Statistics 28. *P* values < 0.05 were considered statistically significant. Continuous variables were compared using a t-test or Mann-Whitney U-test and categorical variables with the Chi-square test. Logistic regression was used to calculate odds ratios and 95% confidence intervals (CIs). Odds ratios were adjusted (aOR) for maternal age, BMI, smoking, parity, ethnicity (two categories, Caucasian and other), and in vitro fertilization (IVF)/intracytoplasmic sperm injection (ICSI).

## Results

The analysis included a total of 4697 individuals with GDM and 18 492 controls who met the eligibility criteria. Women with GDM were older, had higher pre-pregnancy BMI, were more often multiparous, smoked more frequently during pregnancy and had more often pre-eclampsia than controls (Table 1). In the GDM group, 7.4% of women commenced insulin therapy during pregnancy. Median values of PAPP-A and fβ-hCG MoM were 1.03 and 0.95 in women with GDM and 1.11 (*p* < 0.001) and 0.98 (*p* < 0.001) in controls, respectively. There were statistical differences in the rates of SGA infants (2.6% vs. 3.3% *p* = 0.011), LGA infants (4.5% vs. 1.8% *p* < 0.001), and mean birthweights (3573 g vs. 3488 g, *p* < 0.001) between the GDM and control groups, respectively.

**Table 1:** Maternal characteristics and the perinatal outcomes

	GDM (*n* = 4697)	Controls (*n* = 18 492)	*p*-value
**Age**,** mean (SD)**	30.9 (5.2)	29.2 (5.1)	< 0.001^a^
**≥ 35 years**	1191 (25.4%)	2983 (16.1%)	< 0.001^b^
**BMI**,** median (IQR 25–75)**	27.4 (23.8–32.2)	23.2 (21.1–26.0)	< 0.001^c^
**Nulliparous**	1721 (36.6%)	7740 (41.9%)	< 0.001^b^
Smoking *	735 (16.2%)	2565 (14.3%)	0.001^b^
**Caucasian**	4544 (96.9%)	18 029 (97.6%)	0.003^b^
**IVF/ICSI**	171 (3.6%)	544 (2.9%)	0.013^b^
**Insulin started during pregnancy**	346 (7.4%)	− (0.0%)	< 0.001^b^
**First trimester screening**			
Gestational age blood sample, days (SD)	75.9 (SD 5.8)	76.2 (SD 5.7)	0.002
PAPP-A MoM median (IQR 25–75)	1.03 (0.70–1.50)	1.11 (0.76–1.61)	< 0.001^c^
Fβ-hCG MoM median (IQR 25–75)	0.95 (0.64–1.41)	0.98 (0.67–1.46)	< 0.001^c^
NT mm median (IQR 25–75)	1.20 (1.0-1.4)	1.20 (1.0-1.4)	< 0.001^c^
**Pre-eclampsia**	127 (2.7%)	376 (2.0%)	0.005^b^
**Gestational age at the delivery median**,** (IQR 25–75)**	39.4 (38.6–40.4)	39.9 (39.0-40.7)	< 0.001^c^
**Birth weight g mean (SD)**	3573 (524)	3488 (529)	< 0.001^a^
**SGA**	121 (2.6%)	610 (3.3%)	0.011^b^
**LGA**	212 (4.5%)	329 (1.8%)	< 0.001^b^

a = t-test

b = Chi-square -test

c = Mann-Whitney U test

* Data missing *n* = 648

Abbreviations: BMI, body mass index; IQR, interquartile range; GDM, gestational diabetes; NT, nuchal translucency; PAPP-A, pregnancy associated plasma protein-A; Fβ-hCG, free beta human chorionic gonadotropin; SGA, small for gestational age; LGA, large for gestational age; NICU; neonatal intensive care unit

In the low PAPP-A MoM ≤ 5th pc group, the proportion of SGA infants was higher compared to the > 5th pc group, in both the GDM group (5.7% vs. 2.4%, *p* < 0.001) and the control group (10.1% vs. 3.0%, *p* < 0.001) (Table 2). Similar patterns were observed in the ≤ 10th pc and > 10th pc groups. No significant variances in the occurrence of LGA infants were observed about elevated PAPP-A MoM levels, either within the GDM or the control group. Association with SGA and LGA in different PAPP-A MoM levels in the overall population was represented in Supplementary Table 1.

**Table 2:** SGA, AGA and LGA together and separately associations with PAPP-A MoM levels within GDM and control groups

			GDM *n* = 4697		
**PAPP-A MoM**		**SGA**	**AGA**	**LGA**	*p*-value^a^
≤ 5th pc (0.42)	*n* = 279	16 (5.7%)	256 (91.8%)	7 (2.5%)	< 0.001
> 5th pc	*n* = 4418	105 (2.4%)	4108 (93.0%)	205 (4.6%)	
*p*-value^a^		*p* < 0.001	ref.	*p* = 0.117	
≤ 10th pc (0.53)	*n* = 545	25 (4.6%)	506 (92.8%)	14 (2.6%)	< 0.001
> 10th pc	*n* = 4152	96 (2.3%)	3858 (92.9%)	198 (4.8%)	
*p*-value^a^		*p* < 0.001	ref.	*p* = 0.025	
≥ 90th pc (2.21)	*n* = 407	11 (2.7%)	380 (93.4%)	16 (3.9%)	0.830
< 90th pc	*n* = 4290	110 (2.6%)	3984 (92.9%)	196 (4.6%)	
*p*-value^a^		*p* = 0.883	ref.	*p* = 0.557	
≥ 95th pc (2.69)	*n* = 209	6 (2.9%)	194 (92.8%)	9 (4.3%)	0.954
< 95th pc	*n* = 4488	115 (2.6%)	4170 (92.9%)	203 (4.5%)	
*p*-value^a^		*p* = 0.787	ref.	*p* = 0.890	
			**Controls ** *n* ** = 18 492**		
		**SGA**	**AGA**	**LGA**	
≤ 5th pc (0.42)	*n* = 864	87 (10.1%)	768 (88.9%)	9 (1.0%)	0.021
> 5th pc	*n* = 17 628	523 (3.0%)	16 785 (95.2%)	320 (1.8%)	
*p*-value^a^		*p* < 0.001	ref.	*p* = 0.148	
≤ 10th pc (0.53)	*n* = 1749	130 (7.4%)	1602 (91.6%)	17 (1.0%)	0.010
> 10th pc	*n* = 16 743	480 (2.9%)	15 951 (95.3%)	312 (1.9%)	
*p*-value^a^		*p* < 0.001	ref.	*p* = 0.013	
≥ 90th pc (2.21)	*n* = 1918	41 (2.1%)	1841 (96%)	36 (1.9%)	< 0.001
< 90th pc	*n* = 16 574	569 (3.4%)	15 712 (94.8%)	293 (1.8%)	
*p*-value^a^		*p* = 0.003	ref.	*p* = 0.790	
≥ 95th pc (2.69)	*n* = 958	17 (1.8%)	926 (96.7%)	15 (1.6%)	< 0.001
< 95th pc	*n* = 17 534	593 (3.4%)	16 637 (94.8%)	314 (1.8%)	
*p-value* ^*a*^		*p* = 0.006	ref.	*p* = 0.564	

a = chi square −test

Abbreviations: GDM, Gestational diabetes; AGA, appropriate for gestational age; LGA, large for gestational age; SGA, small for gestational age; pc, percentile; ref., reference

In the control group, a low fβ-hCG MoM level of ≤ 5th pc was associated with a higher prevalence of SGA infants compared to the > 5th pc group (7.3% vs. 3.1%, *p* < 0.001). Similar findings were observed in the ≤ 10th pc and > 10th pc groups, with rates of 6.0% vs. 3.0% (*p* < 0.001), respectively. However, these differences were statistically insignificant in the GDM group (Table 3). In the GDM group, a high fβ-hCG MoM level ≥ 90th percentile was associated with a higher prevalence of LGA infants compared to < 90th pc (6.6% vs. 4.3%, *p* = 0.031), but there was no difference in GDM groups with different cutoffs.

**Table 3:** SGA, AGA and LGA association together and separately with fβ-hCG MoM levels within the GDM and control groups

			GDM *n* = 4697		
		**SGA**	**AGA**	**LGA**	*p*-value^a^
**Fβ-hCG MoM**					
≤ 5th pc (0.39)	*n* = 262	11 (4.2%)	244 (93.1%)	7 (2.7%)	0.085
> 5th pc	*n* = 4435	110 (2.5%)	4120 (92.9%)	205 (4.6%)	
*p*-value^a^		*p* = 0.101	ref.	*p* = 0.153	
≤ 10th pc (0.47)	*n* = 528	15 (2.8%)	487 (92.2%)	26 (4.9%)	0.813
> 10th pc	*n* = 4169	106 (2.5%)	3877 (93.0%)	186 (4.5%)	
*p*-value^a^		*p* = 0.670	ref.	*p* = 0.619	
≥ 90th pc (2.07)	*n* = 441	9 (2.0%)	403 (91.4%)	29 (6.6%)	0.072
< 90th pc	*n* = 4256	112 (2.6%)	3961 (93.1%)	183 (4.3%)	
*p*-value^a^		*p* = 0.500	ref.	*p* = 0.031	
≥ 95th pc (2.60)	*n* = 226	4 (1.8%)	207 (91.6%)	15 (6.6%)	0.221
< 95th pc	*n* = 4471	117 (2.6%)	4157 (93.0%)	197 (4.4%)	
*p*-value^a^		*p* = 0.461	ref.	*p* = 0.123	
			**Controls ** *n* ** = 18 492**		
		**SGA**	**AGA**	**LGA**	*p-value* ^*a*^
≤ 5th pc (0.39)	*n* = 896	65 (7.3%)	818 (91.3%)	13 (1.5%)	< 0.001
> 5th pc	*n* = 17 596	545 (3.1%)	16,735 (95.1%)	316 (1.8%)	
*p-value* ^*a*^		*p* < 0.001	ref.	*p* = 0.545	
≤ 10th pc (0.47)	*n* = 1790	108 (6.0%)	1662 (92.9%)	20 (1.1%)	< 0.001
> 10th pc	*n* = 16,711	502 (3.0%)	15,891 (95.1%)	309 (1.9%)	
*p-value* ^*a*^		*p* < 0.001	ref.	*p* = 0.037	
≥ 90th pc (2.07)	*n* = 1873	52 (2.8%)	1783 (95.2%)	38 (2.0%)	0.291
< 90th pc	*n* = 16 619	558 (3.4%)	15,770 (94.9%)	291 (1.8%)	
*p-value* ^*a*^		*p* = 0.188	ref.	*p* = 0.408	
≥ 95th pc (2.60)	*n* = 927	22 (2.4%)	888 (95.8%)	17 (1.8%)	0.269
< 95th pc	*n* = 17 576	588 (3.3%)	16,665 (94.9%)	312 (1.8%)	
*p-value* ^*a*^		*p* = 0.106	ref.	*p* = 0.929	

a = chi square −test

Abbreviations: GDM, Gestational diabetes; AGA, appropriate for gestational age; LGA, large for gestational age; SGA, small for gestational age; pc, percentile; ref., reference

In the low PAPP-A MoM ≤ 5th pc group, the adjusted odds ratios (aORs) for SGA infants were 2.7 (95% CI 1.5–4.7) in the GDM group and 3.8 (95% CI 3.0–4.9) in the control group (Table 4). In the low PAPP-A MoM ≤ 10th pc group, the aOR for the GDM was 2.2 (95% CI 1.4–3.5), and in the control group, it was 2.8 (95% CI 2.3–3.5). The only differences in the aOR for LGA infants in PAPP-A MoM groups were in the low PAPP-A ≤ 10th pc group, where the aOR for LGA was 0.5 (95% CI 0.3–0.9) in the GDM and 0.5 (95% CI 0.3–0.8) in the control group. OR for SGA and LGA in different PAPP-A MoM levels and overall, with GDM was represented in Supplementary Table 2.

**Table 4:** Adjusted odds ratios (aORs) for SGA and LGA in different PAPP-A and fβ-hCG groups

			GDM						Controls		
**PAPP-A MoM**	SGA aOR	95% CI	AGA	LGA aOR	95% CI		SGA aOR	95% CI	AGA	LGA aOR	95% CI
≤ 5th pc (0.42)	2.7	1.5–4.7	ref.	0.5	0.3–1.1		3.8	3.0–4.9	ref.	0.5	0.3–1.1
≤ 10th pc (0.53)	2.2	1.4–3.5	ref.	0.5	0.3–0.9		2.8	2.3–3.5	ref.	0.5	0.3–0.8
≥ 90th pc (2.21)	1.0	0.5–1.9	ref.	1.0	0.5–1.9		0.6	0.4–0.8	ref.	1.1	0.7–1.5
≥ 95th pc (2.69)	1.1	0.5–2.6	ref.	1.1	0.5–2.6		0.5	0.3–0.8	ref.	0.9	0.5–1.5
**β-hCG MoM**											
≤ 5th pc (0.39)	1.5	0.8–3.0	ref.	0.5	0.2–1.2		2.3	1.8–3.1	ref.	0.8	0.4–1.4
≤ 10th pc (0.47)	1.1	0.6–1.9	ref.	1.0	0.6–1.6		2.0	1.6–2.5	ref.	0.6	0.4–0.9
≥ 90th pc (2.07)	0.8	0.4–1.6	ref.	1.6	1.1–2.5		0.8	0.6–1.1	ref.	1.2	0.9–1.8
≥ 95th pc (2.60)	0.7	0.2–1.9	ref.	1.6	0.9–2.8		0.7	0.5–1.1	ref.	1.1	0.6–1.8

Abbreviations: GDM, Gestational diabetes; AGA, appropriate for gestational age; LGA, large for gestational age; SGA, small for gestational age; aOR, odds ratio adjusted for maternal age, BMI, ethnicity, IVF/ICSI, parity and smoking; pc, percentile; ref., reference

In the control group, the aORs for SGA infants in ≤ 5th and ≤ 10th pc fβ-hCG MoM groups were 2.3 (95% CI 1.8–3.1) and 2.0 (95% CI 1.6–2.5), respectively. There were no statistical differences according to low fβ-hCG in the GDM group. In the GDM group, the high fβ-hCG MoM group with ≥ 90th pc had an aOR of 1.6 (95% CI 1.1–2.5) for LGA infants, but no statistically significant differences were observed in other groups or levels.

## Discussion

This was the first population-based study that showed low PAPP-A MoM levels associated with an increased risk of SGA infants both in GDM and non-GDM pregnancies. However, this association was not as notable in the GDM group as in the control group. High PAPP-A MoM levels, instead, showed no association with LGA infants, regardless of the presence of GDM. Low fβ-hCG MoM levels were significantly associated with SGA infants only in the control group.

Previous studies have consistently identified low PAPP-A MoM levels as a risk factor for SGA, which aligns with the findings of this study [22, 23, 25, 42, 43]. Our study further extends this finding in women with GDM, although the risk appears to be lower than in the non-GDM group (Supplementary Table 2). Notably, this is the first study to establish this association in GDM patients. Additionally, our findings suggest that low PAPP-A levels have a greater impact on fetal growth than potentially abnormal maternal glucose metabolism, even though this was not possible to evaluate further in this study. Maternal weight gain during pregnancy is also a factor in fetal weight development, however, in an earlier study there was no difference in gestational weight gain between low and normal PAPP-A study groups [15]. In our study, we unfortunately had no information on women’s weight gain during pregnancy. The association between low PAPP-A and SGA in women with GDM should be considered despite GDM is usually associated with the risk of LGA [2, 9]. Our results overall are consistent with the association between low PAPP-A MoM and lower birthweight compared to normal PAPP-A levels [8]. The pathway through which PAPP-A affects fetal growth may be through its role as an enzyme degrading IGFBPs-4 and − 5 which are mitogenic and antiapoptotic endocrine factors essential to placental function and fetal growth. Moreover, it has been suggested that PAPP-A levels may have further impact on the growth of the offspring also after birth [27].

Earlier study has shown the correlation between PAPP-A levels and birthweight [44]. It can be hypothesized that high PAPP-A values could lead to increased growth. In our study, elevated PAPP-A MoM levels were not associated with LGA. However, previous studies have reported contrary results [24, 26, 45, 46]. Wells et al. found a positive association between PAPP-A MoM and birthweight. In their study, the highest quartile of PAPP-A MoM had a higher prevalence of LGA infants compared to the lowest quartile, with an OR of 2.2 (1.4–3.5). They suggested that high PAPP-A levels might be useful in identifying LGA pregnancies, regardless of GDM status [10]. This result can be explained by a different study design and higher prevalence of SGA in low PAPP-A MoM levels than in high ones. Another study found that extremely high PAPP-A MoM values (above the 99th percentile) were associated with LGA infants [25]. However, the FASTER trial did not find a significant association between high PAPP-A levels and adverse pregnancy outcomes, including macrosomia [23]. In our study, high PAPP-A MoM levels in the GDM group were not associated with an increased risk of LGA infants. In the control group, higher PAPP-A MoM values were associated with a lower prevalence of SGA, but there was no significant difference in LGA. According to our findings, high PAPP-A MoM appears to be a protective factor against SGA in pregnancies without GDM and does not independently increase the risk of LGA, regardless of GDM status. However, in the GDM group, the prevalence of LGA infants was higher at every PAPP-A MoM level compared to the control group, which may be attributed to the mother’s glucose metabolism.

The FASTER trial reported a correlation between fβ-hCG MoM ≤ 5th pc and birthweight below the 10th pc (aOR 1.6, 95% CI 1.3–1.8) [23]. Two previous studies observed that low fβ-hCG MoM levels are linket to SGA, and low birthweight, which aligns with our findings [30, 32]. In the study by Sirikunalai et al., no association was found between low fβ-hCG MoM and GDM [32]. In our study, low fβ-hCG MoM showed an increased risk for SGA only in the control group (aOR 2.0–2.3, 95% CI 1.6–3.1), and the risk for LGA was not found in either the GDM or the control group. However, there is a contradictory study where low fβ-hCG MoM was not associated with SGA, but study methods and population with overrepresentation of LGA were different [25].

Other studies have shown that fβ-hCG levels greater than the 95th pc or over 2 MoM were associated with SGA, with a relative risk (RR) of 2.7 (95% CI 2.1–3.5). [25, 35] In our study, we observed that the prevalence of SGA did not vary with high fβ-hCG MoM levels, whether GDM was present or not. However, within the GDM group, an elevated fβ-hCG MoM level beyond the 90th pc exhibited an association with LGA (aOR 1.6, 95% CI 1.1–2.5), while surpassing the 95th pc did not reach statistical significance. Among the control group, there was no difference in LGA rates based on high fβ-hCG MoM levels. Additionally, in a previous study, there was no difference in LGA rates between the GDM and control groups based on fβ-hCG MoM levels [24].

The study’s strengths included a large population-based sample size of GDM patients and controls, as well as reliable register data. The definitions of SGA and LGA were clear and well-defined, and the study design allowed for a precise comparison of these newborn size categories. However, there were some limitations, such as OGTT was not performed for all pregnant women as a universal screening method; low risk women were excluded from screening as described in the Methods section according to Finnish Current Care guidelines [38]. Also, reliable data on oral glucose medication were not available and the classification of the GDM severity or information on women’s gestational weight gain was not available due to the study type. In this study, we did not evaluate the association of pre-eclampsia and SGA.

## Conclusion

This study was the first to establish an association between low PAPP-A MoM levels and the risk of SGA in patients who were diagnosed with GDM subsequently during pregnancy. Association with low PAPP-A MoM and SGA is present despite of GDM status but seems to be weaker in the GDM group. In this study, high PAPP-A MoM levels appeared to be a protective factor against SGA, but they were not found to be a risk factor for LGA, regardless of GDM status. In non-GDM pregnancies, low fβ-hCG MoM levels were associated with SGA, but this association was not observed in GDM patients. Conversely, a high fβ-hCG MoM level ≥ 90th pc was associated with LGA only in GDM pregnancies. These findings supplement the knowledge of the role of PAPP-A as an important part of fetal growth.

## Electronic supplementary material

Below is the link to the electronic supplementary material.

Supplementary Material 1

## Data Availability

No datasets were generated or analysed during the current study.

## Abbreviations

PAPP-A: Pregnancy associated plasma protein-A
MoM: multiples of the median
DM: diabetes mellitus
GDM: gestational diabetes mellitus
