# Supplementary material for: First trimester maternal serum PAPP-A and free β-hCG levels and risk of SGA or LGA in women with and without GDM
Source: BMC Pregnancy Childbirth. 2024 Sep 6;24:580. doi: 10.1186/s12884-024-06786-4 (PMC11380344; doi:10.1186/s12884-024-06786-4)
Supplement: Supplementary file 1 — Supplementary Material 1 [file 12884_2024_6786_MOESM1_ESM.docx]

| Supplementary table 1. SGA and LGA associations with PAPP-A MoM levels with overall population | | | | | | |  |
| --- | --- | --- | --- | --- | --- | --- | --- |
|  |  |  |  |  |  |  |  |
| PAPP-A MoM | |  | SGA | AGA | LGA |  | *p*-value^a^ |
|  |  |  |  |  |  |  |  |
| ≤ 5th pc (0.42) | |  | 103 (9.0%) | 1024 (89.6%) | 16 (1.4%) |  | <0.001 |
| > 5th pc |  |  | 628 (2.8%) | 20 893 (94.8%) | 525 (2.4%) |  |  |
| *p*-value^a^ |  |  | <0.001 | ref. | 0.061 |  |  |
|  |  |  |  |  |  |  |  |
| ≤ 10th pc (0.53) | |  | 155 (6.8%) | 2108 (91.9%) | 31 (1.4%) |  | <0.001 |
| > 10th pc |  |  | 576 (2.8%) | 19 809 (94.8%) | 510 (2.4%) |  |  |
| *p*-value^a^ |  |  | <0.001 | ref. | 0.002 |  |  |
|  |  |  |  |  |  |  |  |
| ≥ 90th pc (2.21) | |  | 52 (2.2%) | 2221 (95.5%) | 52 (2.2%) |  | 0.027 |
| < 90th pc |  |  | 679 (3.3%) | 19 696 (94.4%) | 489 (2.3%) |  |  |
| *p*-value^a^ |  |  | 0.008 | ref. | 0.691 |  |  |
|  |  |  |  |  |  |  |  |
| ≥ 95th pc (2.69) | |  | 23 (2.0%) | 1120 (96.0%) | 24 (2.1%) |  | 0.047 |
| < 95th pc |  |  | 708 (3.2%) | 20 797 (94.4%) | 517 (2.3%) |  |  |
| *p*-value^a^ |  |  | 0.017 | ref. | 0.481 |  |  |
|  |  |  |  |  |  |  |  |
| ^a = chi x2 -test^ |  |  |  |  |  |  |  |
| ^Abbreviations: GDM, Gestational diabetes; AGA, appropriate for gestational age; LGA, large for gestational age; SGA, small for gestational age; pc, percentile; ref., reference^ | | | | | | | |

| Supplementary table 2. Odds ratio for SGA and LGA with GDM versus without GDM overall and in different PAPP-A MoM levels | | | | | |
| --- | --- | --- | --- | --- | --- |
|  |  |  | GDM |  |  |
| PAPP-A MoM | SGA OR | 95% CI | AGA | LGA OR | 95% CI |
|  |  |  |  |  |  |
| Overall | 0.798 | 0.655-0.972 | ref. | 2.592 | 2.174-3.090 |
|  |  |  |  |  |  |
| ≤ 5th pc (0.42) | 0.552 | 0.318-0.958 | ref. | 2.333 | 0.860-6.329 |
|  |  |  |  |  |  |
| ≤ 10th pc (0.53) | 0.609 | 0.392-0.945 | ref. | 2.607 | 1.276-5.327 |
|  |  |  |  |  |  |
| ≥ 90th pc (2.21) | 1.300 | 0.662-2.552 | ref. | 2.153 | 1.183-3.920 |
|  |  |  |  |  |  |
| ≥ 95th pc (2.69) | 0.780 | 0.266-2.288 | ref. | 3.785 | 1.860-7.704 |
|  |  |  |  |  |  |
| ^Abbreviations: GDM, Gestational diabetes; AGA, appropriate for gestational age; LGA, large for gestational age; SGA, small for gestational age; pc, percentile; ref., reference^ | | | | | |
